# Supplementary material for: Activity-dependent CO2 production in the axon triggers opening of Connexin32 in the Schwann cell paranode
Source: eLife. 2026 Jul 6;14:RP107085. doi: 10.7554/eLife.107085 (PMC13336771; doi:10.7554/eLife.107085)
Supplement: Peer review history

# Editor report: eLife Assessment

**DOI:** 10.7554/eLife.107085.3.sa0

**Authors:** Kenton J Swartz

**Affiliations:** National Institute of Neurological Disorders and Stroke United States

**Keywords:** Convincing, Important

This manuscript describes **convincing** and very interesting findings that substantially advance our understanding of a major research question on the role of Cx32 hemichannels in the Schwann cell paranode. It provides an interdisciplinary integration of imaging, in silico approaches, and functional data. This **important** study proposes a new mechanism with profound physiological relevance and provides new insights into glial modulation of electrical conduction in sensory/motor myelinated nerves.

# Referee report: Reviewer #1 (Public review)

**DOI:** 10.7554/eLife.107085.3.sa1

The manuscript by Butler et al. explores a novel physiological role for connexin 32 (Cx32) hemichannels in Schwann cells of peripheral nerves. Building on the authors' prior work on CO_2_-sensitive gating of connexin hemichannels, this study proposes that axonal activity-dependent mitochondrial CO_2_ production promotes the opening of Cx32 hemichannels in adjacent Schwann cells, a process regulated by carbonic anhydrase (CA) activity and AQP1. This work reveals a new form of intercellular communication that may contribute to the regulation of conduction velocity.

The authors aimed to determine whether CO_2_ acts as an activity-dependent signal in peripheral nerves through activation of Cx32 hemichannels in myelinating Schwann cells. The study is strengthened by the use of complementary techniques, including in silico approaches, pharmacological manipulation, dye uptake assays, calcium imaging, adenoviral delivery of dominant-negative Cx32 constructs targeted to Schwann cells, and extracellular recordings in isolated sciatic nerves. Together, these methods allow the authors to connect molecular mechanisms with tissue-level function.

The study has a few technical limitations, and some aspects of the interpretation require caution. Limitations in antibody specificity complicate interpretation of the precise distribution of the signaling pathway components studied here. Dye uptake into the outer myelin layer is consistent with hemichannel opening, but it does not by itself prove that Cx32 directly mediates the observed permeability changes. Similarly, Ca^2+^ signals associated with Cx32 activation could reflect direct Ca^2+^ permeability through Cx32 or secondary activation of other Ca^2+^ entry or release pathways. Finally, hemichannel opening is assessed primarily using FITC uptake, which may not fully capture the complexity of Cx32 gating or distinguish between different conductive states.

Overall, the authors provide substantial evidence that activity-dependent CO_2_ production can influence Schwann cells through a pathway involving CA, AQP1, and Cx32. The results support the broad conclusions of the study, although some direct mechanistic links require further validation. The work is likely to have an important impact because it proposes a novel role for CO_2_ as a local signaling molecule in peripheral nerves and may provide new insight into how Schwann cells detect axonal activity and regulate peripheral nerve physiology.

Comments on revised version.

The authors have addressed all of my concerns. The manuscript is now much improved and reads very well. Congrats to all the research team.

# Author comment: Author response

**DOI:** 10.7554/eLife.107085.3.sa2

**Authors:** Jack Butler, Lowell Mott, Amol Bhandare, Angus Brown, Nicholas Dale

**Affiliations:** University of Warwick Coventry United Kingdom; University of Warwick Coventry United Kingdom; University of Warwick Coventry United Kingdom; University of Nottingham Nottingham United Kingdom; University of Warwick Coventry United Kingdom

The following is the authors’ response to the original reviews.

**Public Reviews:**

**Reviewer #1 (Public review):**

The manuscript by Butler et al. explores a novel physiological role for connexin 32 (Cx32) hemichannels in Schwann cells at peripheral nerves. Building on the authors' prior work on CO_2_ - sensitive gating of connexins, this study proposes that mitochondrial CO_2_ production dependent on neuronal activity promotes the opening of Cx32 hemichannels in the paranode, which in turn modulates neuronal activity by reducing conduction velocity. This hypothesis is addressed using a multifaceted approach that includes immunofluorescence microscopy, dye uptake assays, calcium imaging, computational modeling, and extracellular recordings in isolated sciatic nerves.

Among the strengths of the study are the interdisciplinary integration of imaging, in silico approaches, and functional data. Also, this study proposes a new mechanism with profound physiological relevance. Specifically, Butler et al. provide new insights into glial modulation of electrical conduction in sensory/motor myelinated nerves.

In the current state, the study has some limitations. The evidence linking Cx32 to the observed dye uptake and conduction velocity changes relies primarily on pharmacological inhibition with carbenoxolone, which lacks specificity. The imaging data show overlapping marker signals that preclude the anatomical distinction between nodes and paranodes. FITC uptake, while convincing to test Cx32 hemichannel gating, lacks spatial-temporal information and validation of distribution and localization to viable intracellular compartments. Moreover, while the findings are intriguing, functional proof that Cx32 regulates conduction velocity through ATP release or other downstream effects remains incomplete. Further work using targeted genetic tools, live-tissue imaging, and additional controls would strengthen the mechanistic conclusions.

Overall, the manuscript offers compelling preliminary evidence that supports a new role for Cx32 in peripheral nerve physiology and raises important questions for future investigation.

We thank the reviewer for their comments and agree that the evidence for involvement of Cx32 is indirect. We have now used viral expression of Cx32^DN^ in SCs to remove CO_2_ sensitivity from the endogenous Cx32 to strengthen this link. We have reviewed our presentation of the morphology in terms of the node/paranode/juxtaparanode distribution and adjusted accordingly. We have added new data using GCaMP transduced into Schwann cells that provides the live-tissue imaging that the reviewer requests.

**Reviewer #2 (Public review):**

Summary:

This article aims to demonstrate that local production of CO_2_ at the axonal node opens Cx32 hemichannels in the Schwann cell paranode, and that CO_2_ diffuses through the AQP1 channel to reach Cx32 and trigger its opening. The authors also present evidence supporting a physiological role for this regulatory mechanism. They propose that CO_2_-dependent Cx32 activation mediates activity-dependent Ca^2+^ influx into the paranode, and by increasing the leak current across the myelin sheath, it contributes to a slowing of action potential conduction velocity.

The study presents a very interesting and novel mechanism for the physiological regulation of Cx32 hemichannels. The findings are relevant to the field, and the methods and results are of good quality, with some improvements in interpretation and explanation required, and some minor experimental suggestions.

Strengths:

The article is solid in terms of the novelty of the findings and relevance for the physiology of myelinated axons. In addition, it is of major interest for the Connexin field because it explores a physiological way to open Cx32 hemichannels. The experiments are well elaborated, and most of them are sufficient for the main points described by the authors. The finding that nervous activity will trigger the mechanism of hemichannel opening by CO2 is probably the most relevant biological mechanism derived from this article.

Weaknesses:

Throughout the manuscript, the authors interpret their findings as if the described mechanism specifically occurs in the node and paranode regions. However, there is no direct evidence identifying the precise site of CO_2_ production or the activation site of Cx32 hemichannels. Therefore, statements such as the one in the title ("activity-dependent CO_2_ production in the axonal node opens Cx32 in the Schwann cell paranode") should be reconsidered or removed, as they may be misleading and are not essential to the interpretation of the data. In addition, the participation of aquaporin AQP1 as the main conduit for CO2 diffusion through the plasma membrane could have another interpretation.

We thank the reviewer for their comments and agree that we do not have direct evidence for the site of CO_2_ production or the site of activation of Cx32 hemichannels. This direct evidence is extremely difficult to obtain, and we therefore depend on indirect arguments. Mitochondria represent the major source of CO_2_, and their distribution will therefore indicate where CO_2_ is likely to be produced. We agree that this is not essential to the interpretation of the data and have adjusted the text as recommended. We have added a section to the Discussion to consider this point in more detail. The reviewer alludes to a reported interaction between AQP1 and NaV1.8 as a possible alternative interpretation. We can confidently rule this out as the AQP1 blocker has no effect on the compound action potential.

**Recommendations for the authors:**

**Reviewer #1 (Recommendations for the authors):**

Main comments:

(1) While the imaging system used in this study is technically capable of resolving nodes and paranodes, interpretation depends critically on marker specificity and tissue orientation. In some figures, markers such as Caspr or KCNA2 appear to partially overlap with KCNQ2 or the putative axonal node, which could reflect biological proximity but may also result from incomplete spatial separation in the z-dimension or the curvature of teased fibers. Similarly, Cx32 immunoreactivity or FITC signal is occasionally seen within nodal gaps, raising questions about how accurately this data supports the author's hypothesis. Additionally, while the authors claim that AQP1 is localized in nodes, the data suggest the opposite. Clarifying these patterns using fluorescence intensity line scans or additional nodal markers such as Nav1.6 or Ankyrin G would help distinguish overlapping signals from true domain-specific localization and reinforce the spatial conclusions of the study.

We have changed our presentation of the localisation studies. We have concentrated on colocalization of Cx32 and AQP1 (now Fig 2) and moved the other studies to supplements to this figure. While we have retained the same images of Cx32 and AQP1 localisation, we have emphasized that these are SIM images and thus higher resolution than conventional LSM images, and also from a single optical plane. We have also clarified that the colocalization studies are restricted to analysis of the node/paranode regions.

(2) To strengthen the conclusion that Cx32 specifically mediates the observed dye uptake, additional data or an alternative approach would be valuable. One feasible, though technically demanding, strategy would be the use of AAV-mediated delivery of Cx32-targeting shRNA directly into the sciatic nerve, ideally under a Schwann cell-specific promoter. This approach could achieve localized, cell-type-specific knockdown of Cx32 within a relevant time frame. Alternatively, the authors are encouraged to consider using additional pharmacological inhibitors to exclude the contribution of other conduction pathways, such as pannexin channels. These complementary strategies would reduce the interpretive ambiguity associated with non-specific blockade.

We agree that this is desirable and have used Cx32^DN^ under the control of the Mpz promoter (delivered by AAV via intranerval injection). This approach has several advantages -the Cx32^DN^ subunit coassembles with endogenous Cx32^WT^ and the heteromeric assemblies lack CO_2_ sensitivity (first shown in Butler & Dale, 2023; and this strategy used with Cx26 to demonstrate its role in the control of breathing van de Wiel, 2020). This is a new figure (Fig 9). We have included supplemental figures with Fig 9 to document the coassembly of Cx32^DN^ with Cx32^WT^ by FRET.

These new data test a very specific hypothesis: that CO_2_ binding to Cx32 is responsible for the CO_2_ sensitivity of the nerve. We find by comparing transduced and non-transduced fibres in the same nerve that Cx32^DN^ essentially abolishes activity dependent loading of FITC into the Schwann cells.

(3) Related to FITC experiments: Assuming the hypothesis of the authors is correct and CO2 release is restricted to the node, one should expect that if the major source of CO2 is in the nodal mitochondria, the hemichannels adjacent to the node will open first, assuming the spatial-temporal diffusion of CO2. To demonstrate this point, I would strongly suggest performing tissue imaging with real-time dye uptake. This approach should capture the FITC wave starting from the Cx32 channel opening in the paranode, as expected. Visualization of uptake in fixed and sectioned tissue is not the ideal approach to detect functional hemichannel opening in intact, viable cells, and at this point, they do not demonstrate that the uptake occurs in the node. From my perspective, if real-time experiments using isolated axons are feasible, it would make this paper more solid.

The suggested method is not practical as the FITC in solution will be fluorescent and thus obscure the entry of FITC into the paranode. We have however expressed GCaMP8 under the control of the Mpz promoter, and this is expressed at paranodes and gives a CO_2_ and activity-dependent Ca^2+^ signal at the paranode. This gives a real time measure of the effect of CO_2_ on the nerve. The GCaMP8 signal is enhanced by AZ and blocked by TC AQP1-1 (see below).

(4) In Figure 5, Supplement 1, the authors present data using GRAB-ATP to suggest that Cx31.3 hemichannels do not release ATP under CO_2_ stimulation. However, control experiments with GRAB-ATP alone (without Cx31.3 expression) are not shown, and parallel conditions with Cx32-expressing cells are lacking. Including these controls would strengthen the manuscript. Finally, testing the permeability of Cx31.3 to FITC directly, using the same conditions as in the main experiments, would clarify whether the discrepancy reflects differences in molecular permselectivity or CO_2_ sensitivity.

Figure 5 supplement 1, does show GRAB_ATP_ alone without Cx31.3 expression (in the box plot). However, we have now added raw traces for this to the figure in panel B. CO_2_-dependent and voltage dependent ATP release via Cx32 has been previously shown in two papers (Butler & Dale 2023, Frontiers Cell Neurosci; Lovatt et al 2025, J Biol Chem). The Cx32^DN^ result (above) further eliminates any contribution of Cx31.3.

(5) Suggestion: It would be valuable to explore whether the proposed mechanism is conserved across both motor and sensory neurons, as this would broaden its physiological relevance. Since the sciatic nerve contains both fiber types, selective analysis or comparative data could clarify whether hemichannel activity is differentially regulated or restricted to a specific neuronal subtype.

This is a great idea, but well beyond the scope of this paper. In an ex vivo preparation it would be very difficult to selectively stimulate the sensory vs motor fibres.

Suggestions to improve data presentation and other minor comments:

(1) Reduce/reorganize the figures to make the paper straightforward. For example, (a) immunofluorescence data showing the CO2 signaling machinery could be represented in one single figure; (b) Figure 1 could include all the findings and keep it as a final figure to summarize what the authors claim.

We thank the reviewer for these suggestions. We prefer to keep Fig 1 up front to have our hypothesis clear for the reader to assist their interpretation as they go through the paper. We have altered the balance of figure supplements and main figures that document the immunolocalisation studies to concentrate on the main areas of novelty (AQP1 and Cx32 colocalisation and CA localisation).

(2) The following phrase in the Results section is incomplete: "There was colocalization between Cx32 and CytC in the Schwann cell paranode, and (Fig 2, mean; 95% confidence interval, M1: 0.314; 0.198, 0.431 and M2: 0.261; 0.165, 0.357)."

We have corrected this

Additionally, the three values for M1 and M2 should be clearly defined and contextualized. In the current state, I couldn't understand them.

The three values are mean and lower and upper 95% confidence limit:

M1: mean 0.314; 95% CI, 0.198 to 0.431

We have now made this clearer in the text.

(3) It is unclear whether the authors calculate Manders' coefficients across the whole image or selectively at the node/paranode. Clarifying this would help interpret the specificity of co-localization claims.

The Manders’ coefficients were selectively calculated at the node/paranode and we have amended the text to clarify this.

(4) It is possible that mislocalization of CytC and SFXN1 could reflect antibody unspecificity or post-isolation alterations in protein distribution (e.g., apoptosis or stress). The authors briefly discussed this observation, but it could be a good idea to consider the use of an additional antibody to validate mitochondria localization.

Apoptosis or stress is unlikely as the isolated nerves were fixed immediately after isolation with little dissection prior to fixation.

The SFXN1 antibody was validated by Fowler et al 2013, and IP-HTMS confirmed SFXN1 as an interacting partner with Cx32. In this paper they also described SFXN1 as being present at the plasma membrane, the speculation being that it was taken there by Cx32.

We think this is probably a valid result and we have further cited the Fowler et al 2013 paper in our discussion of this point.

(5) Figure 4: The legend states: "Arrow heads indicate the node, and arrows depict the outer myelin." However, no arrows are visible in the figure. Please check.

Corrected.

(6) Figure 5: Keep consistency: Include in panel N that trpa1 inhibitor is in the presence of 70mmHg PCO2, as indicated for cbx in the same panel.

Done

(7) Figure 5 Supplement 1: Normalization using 1 concentration of ATP could not be appropriate if the sensor-dependent signal is not linear. If possible, authors should make a concentration-response curve and fit the data using the appropriate equation.

Over the range we are measuring ATP (low µM) GRAB_ATP_ is approximately linear to allow a single point calibration -we documented this in Butler and Dale 2023. This is also shown in the original paper describing GRAB_ATP_ (Wu et al 2022 Neuron). We have clarified this point in the methods by referring to these papers.

(8) Figure 6: The increase in FITC signal could represent a basal uptake over time. Authors should clarify the magnitude/rate of the basal uptake. Another option is showing a picture of the uptake using the control frequency at a time of 10 min. Legend: It is not clear in panel C if this picture corresponds to frequency stimulation. If so, it would be beneficial to specify the time.

Could dye loading in this Fig simply be time dependent rather than stimulation dependent? Our data show that this is not the case -the dye loading controls of Fig 5A were exposed to FITC for 10 mins at 35 mmHg PCO_2_ -very little loading is apparent. We now explicitly make this point in the text. Our use of Cx32^DN^ also eliminates this explanation, by demonstrating the necessity of CO_2_ binding to Cx32 for dye loading to occur.

As there is no panel C in this figure, we assume the referee means panel B and have added the frequency of stimulation and time duration used to achieve the loading.

(9) Please revise the legend of Figure 7. It seems to refer to a previous version of the manuscript's figure.

Thanks for pointing this out. We omitted giving a letter to one of the panels and we have corrected this so that legend and figure now correspond.

(10) Figures 10 and 11. Please consider including a bright field image or indicating with an arrow where the node and/or paranode is located.

The old Fig 11 has been omitted. The old Fig 11 is now Fig 10. Unfortunately, we cannot add a bright field image as we did not save these in this experiment.

(11) Figure 11. The authors could consider doing this experiment in the presence of Cx32 blockers to strengthen their conclusion.

We have decided to remove this figure as it the information it contains is shown in the new GCaMP8 figure (Fig 12).

(12) Figure 12: Calcium signal increases in different areas beyond the ROI. Not clear that the calcium signal is restricted to the node, as shown in previous figures. Please clarify if the preparation is different.

We agree that this is a limitation – there is a lot of out of focus light due to Fluo4 being membrane permeable and loading many fibres within the nerve (potentially both axon and Schwann cell). Importantly, this phenomenon occurs in the in-focus ROI (for which we show BF image).

As we think this is basically a limitation of using Fluo4-AM, we have now produced better data using GCaMP8 under the Mpz promoter (new Fig 12). This expresses at the paranode and in far fewer fibres so the resolution of the recordings is better. We have added these new data into the main body of the paper and relegated the Fluo4 data as a figure supplement to Fig 12 that provides independent supporting information.

(13) Figure 13: Please indicate the stimulation frequency. The authors could consider attaching Figure 7 Supplement 1 to this figure to make the manuscript straightforward.

Frequency now indicated.

With regard to the original Figure 7 supplement 1 -thanks for this suggestion. After consideration, we have split this up and attached it as figure supplements to the relevant figures (Figure 6 and Figure 8). We have added equivalent data to Fig 7 (effect of H_2_O_2_). We think this simplifies presentation for the readers.

(14) Figure 7 Supplement 1 and Figure 8 Supplements: Please indicate trace colors in panel A of these figures. Also, correct the spelling issue in the legend of Figure 8 Supplement 1 (for panel B).

Corrected

(15) Statistical clarifications: The authors should specify which experimental groups were included in some statistical analysis where p-values are reported, but the information about which groups are compared is missing.

Corrected

**Reviewer #2 (Recommendations for the authors):**

(1) Localization of CO_2_ production and Cx32 activation

Throughout the manuscript, the authors interpret their findings as if the described mechanism specifically occurs in the node and paranode regions. However, there is no direct evidence identifying the precise site of CO_2_ production or the activation site of Cx32 hemichannels. Therefore, statements such as the one in the title ("activity-dependent CO_2_ production in the axonal node opens Cx32 in the Schwann cell paranode") should be reconsidered or removed, as they may be misleading and are not essential to the interpretation of the data.

We agree that we have not shown this -and now exercise more caution in the description of the results and discuss this point.

(2) Figures 2 and 3 - Cx32, mitochondria, and AQP1 localization

In Figures 2 and 3, it is difficult to clearly discern the localization of Cx32, mitochondria, and AQP1 in the nodal and paranodal regions. The addition of zoomed-in images and 3D reconstructions (or at least orthogonal views) would greatly help clarify whether these components are indeed localized to the axon or Schwann cell, and whether they are specifically enriched in nodal or paranodal domains. As currently presented, the images suggest that all components of this "triad" are broadly distributed within the cells, not restricted to, nor particularly enriched in, nodal or paranodal areas. This observation further supports the concern raised in point 1.

We have revised our presentation of the localisation more clearly and added a section to the discussion to consider this point more fully. We now explicitly mention that these are SIM images and in a single optical plane, therefore colocalization is genuine. We have also clarified that the calculation of Manders’ coefficients was performed only at the node/paranode regions. However, we accept that these components are distributed more widely than the node/paranode.

(3) Figure 5 - Clarify legend labels

In the graph shown in Figure 5, the legend would benefit from more descriptive labeling of the experimental groups. For clarity, indicate that FCCP was applied alone, and that HCO30031 was co-applied with high PCO_2_, to simplify interpretation for the reader.

Corrected

(4) Additional experiment to block mitochondrial CO_2_ production

An experiment should be added to completely or significantly inhibit mitochondrial CO_2_ production, for example, by combining FCCP treatment with a TCA cycle inhibitor such as fluoroacetate. This would more directly demonstrate that CO_2_ generation is required for hemichannel opening during FCCP treatment. It is important to control for this because FCCP can increase ROS production as a result of compensatory metabolic activity (i.e., increased NADH/FADH_2_ generation). Since Cx32 hemichannels are known to be modulated by ROS, and can also regulate mitochondrial ROS production, it is crucial to distinguish the role of CO_2_ from that of ROS in these experiments.

Thanks for this great comment, as it gave us the idea of linking activity-dependent (rather than FCCP-evoked) gating of Cx32 to the TCA cycle and, as the reviewer says, CO_2_ generation more directly. As fluoroacetate is only effective at inhibiting the TCA cycle in glial cells, we used H_2_O_2_ at 50 µM which is highly effective at blocking aconitase in neurons (Tretter & Adam-Vizi, 2000). This greatly reduced FITC dye loading in response to activity. We now include these data in the paper (Fig 7).

We note that our new data with Cx32^DN^ further establishes the link to CO_2_ as opposed to ROS.

Furthermore, to complement the experiments involving carbonic anhydrase (CA) manipulation, additional controls or mechanistic validation may be necessary to support the conclusions drawn.

We think that our use of Cx32^DN^ greatly strengthens our conclusions that CO_2_ is the messenger from the axon that gates Cx32 in the paranode.

(5) AQP1 and Na^+^ channel interaction - alternative interpretation

It has been reported that AQP1 interacts with voltage-gated Na^+^ channels, influencing action potential generation. For example, in AQP1 knockout mice, current injection-evoked action potentials show a reduced peak inward current, suggesting impaired Nav1.8 function (Zhang et al., J. Biol. Chem., 2010; doi: 10.1074/jbc.M109.090233). This raises the possibility that the observed effects of AQP1 inhibition (e.g., with TC AQP1-1) could also result from altered Na^+^ channel activity, not just impaired CO_2_ transport. I suggest that this alternative interpretation be acknowledged and discussed, as the current data do not rule it out.

While constitutive KO of AQP1 does alter action potential generation in DRGs and an interaction between AQP1 and Nav1.8 has been documented, we do not think that this is a viable alternative interpretation of our data. We have measured the CAP during all our manipulations including the use of TC AQP1-1, and its amplitude is unaltered (see Fig 8 fig supplement 1 and Fig 13D). Our data therefore shows that, in the context of our experiments, application of the AQP1 blocker, TC AQP1-1, does not alter Na^+^ channel activity. The difference between our data and the evidence from AQP1 knock-out may arise from the nature of an acute application of an antagonist (short term effect without changing protein expression) and constitutive knock out, which is likely to have longer term effects. We have added some discussion to address this point (last few lines, Page 9).

(6) Figures 11A and 12C - Add heat map calibration

In Figures 11A and 12C, the changes in Ca^2+^ signals are difficult to interpret. In some areas, color changes appear to occur outside of cellular structures. I recommend including a heat map calibration scale for both figures to facilitate the interpretation of the signal intensity and localization.

We agree that these data are limited by the technique used, and as mentioned above we now have GCaMP8 data that has better resolution and strengthens our conclusions.
